# Drug therapy in patients with Parkinson’s disease

**DOI:** 10.1186/2047-9158-1-10

**Published:** 2012-05-24

**Authors:** Thomas Müller

**Affiliations:** 1 Department of Neurology, St. Joseph Hospital Berlin-Weissensee, Gartenstr. 1, 13088, Berlin, Germany

**Keywords:** Motor symptoms, Parkinson’s disease, non motor features, drug therapy

## Abstract

Parkinson`s disease (PD) is a progressive, disabling neurodegenerative disorder with onset of motor and non-motor features. Both reduce quality of life of PD patients and cause caregiver burden. This review aims to provide a survey of possible therapeutic options for treatment of motor and non motor symptoms of PD and to discuss their relation to each other. MAO-B-Inhibitors, NMDA antagonists, dopamine agonists and levodopa with its various application modes mainly improve the dopamine associated motor symptoms in PD. This armentarium of PD drugs only partially influences the onset and occurrence of non motor symptoms. These PD features predominantly result from non dopaminergic neurodegeneration. Autonomic features, such as seborrhea, hyperhidrosis, orthostatic syndrome, salivation, bladder dysfunction, gastrointestinal disturbances, and neuropsychiatric symptoms, such as depression, sleep disorders, psychosis, cognitive dysfunction with impaired execution and impulse control may appear. Drug therapy of these non motor symptoms complicates long-term PD drug therapy due to possible occurrence of drug interactions, - side effects, and altered pharmacokinetic behaviour of applied compounds. Dopamine substituting compounds themselves may contribute to onset of these non motor symptoms. This complicates the differentiation from the disease process itself and influences therapeutic options, which are often limited because of additional morbidity with necessary concomitant drug therapy.

## Introduction

Parkinson`s disease (PD) is a progressive, disabling neurodegenerative disorder. This disease is characterized by an insidious onset with variable expression of predominant motor, vegetative, sensory and psychopathological symptoms. Ongoing loss of nigral dopaminergic presynaptic neurons with a reduction of about 70–80% striatal dopamine mainly leads to clinical diagnosis due to the occurrence of the main motor symptoms and their dopaminergic response. These motor features are akinesia, tremor and rigidity, sometimes even initially in combination of postural disturbances, which mostly appear later in the course of the disease and do not respond to dopaminergic stimulation. Therapeutic approaches of non motor features gain increasing importance in addition to motor symptoms control to improve quality of life in PD patients and their caregivers [1]. Long term treatment of this array of symptoms with various drugs causes the occurrence of short - and long term side effects. Course of PD, expression of motor and non motor symptoms, efficacy and tolerability of therapeutic interventions vary from one patient to another. Therefore an individualized therapeutic regime is performed with repeated control and titration by the treating physician in close cooperation with the patient and his caregiver in clinical practice.

### Treatment of motor symptoms

Mainly akinesia, rigidity and clinical associated features and to a lesser extent tremor respond to dopaminergic stimulation in PD patients. Table 1 provides a proposal for a treatment cascade of dopamine system influencing compounds for PD patients with probable long necessary dopamine substitution therapy following diagnosis.

**Table 1:** Treatment cascade of current dopaminergic substitution tools with respect to the concept of continuous dopaminergic stimulation

Drug	Step	Mode of action within the dopaminergic system	Tolerability	Main clinical relevant side effects	Efficacy
MAO-B-I	I	stabilize dopamine levels in the striatal synaptic cleft by inhibition of dopamine metabolism	+++	risk for rise of raised blood pressure and increase of liver enzymes, contraindication for simultaneous fluoxetine and fluvoxamine use, precaution with application of SSRI in general	+
NMDA-A	I	indirect dopaminergic modulation, reduce motor complications (?)	+	oedema, insomnia, hallucinations	+
DA	II	stimulate directly postsynaptic striatal receptors linked to motor symptom control	+	Orthostatic syndrome, oedema, nausea, slow titriation necessary	++
LD/DDI/COMT-I	III	precursor of dopamine, DDI and COMT-I reduce LD metabolism	+++	orthostatic syndrome, homocysteine elevation (LD/DDI alone), motor complications, diarrhea (COMT-I)	+++
infusion systems (apomorphine, LD)	IV	See DA, respectively LD line	+	Subcutaneous local inflammatory reactions	+++
DBS	V	electric stimulation of the subthalamic nuclei or globus pallidus	+	Social adjustment problems, depression, cognitive dysfunction.	+++

DBS = deep brain stimulation, MAO-B-I = MAO-B-Inhibitors, NMDA-A = NMDA-antagonist, DA = dopamine agonist, LD = levodopa, DDI = decarboxylase inhibitor, COMT-I = inhibitor of catechol-O-methyltransferase, judgement on tolerability and efficacy are based on the personal experience of the author.

### Monoaminooxidase B (MAO-B) inhibition

MAO-B-I stabilize the dopamine levels in the synaptic cleft. Two compounds of the propargylamine group, selegiline and rasagiline, both irreversible MAO-B inhibitors, have demonstrated a symptomatic effect in PD patients. MAO-B-inhibition catalyses the oxidative deamination of active amines and therefore causes prolonged dopamine activity. Selegiline and rasagiline are relative specific inhibitors of MAO-B activity. However this selectivity gets lost at higher drug doses, i.e. selegiline > 20 mg/day and rasagiline > 2 mg/day. These dosages also inhibit MAO-A, which converts other amines, like norepinephrine. Therefore, although low, there is a risk of tyramine-induced hypertension, which is called the "cheese effect", at higher doses by these agents. These compounds are also known to enhance the activity of catecholaminergic neurons by mechanisms other than MAO-B inhibition [2]. Other pharmacological activities such as effect on mitochondrial membrane potential activity, anti-apoptotic and antioxidant efficacy may explain potential neuroprotective mechanisms seen in the laboratory. Correspondingly, clinical trials investigated this putative benign influence on the course of PD [2].

### MAO-B-I and progression of PD

The DATATOP study was a prospective, randomised, double-blind, placebo-controlled study that included 800 patients with PD. After randomisation to either selegiline, α-tocopherol (vitamin E), a combination of both or placebo, the patients were followed up with no other treatment until clinical deterioration calling for initiation of symptomatic therapy with Levodopa (LD) plus dopadecarboxylase inhibitor (DDI) as clinical endpoint. Selegiline, but not α-tocopherol, resulted in a significant delay for LD/DDI requirement compared with placebo (26 versus 15 months; p < 0.0001). However, this beneficial effect of selegiline vanished in the further follow-up investigations of the trial. The main limitation of this study was the potential confounding symptomatic effect of selegiline on the results. Further trials examined the disease modifying potential of rasagiline. Both double-blind, parallel group, randomised, delayed-start clinical studies, the TEMPO and the ADAGIO trial, included early PD patients. They were randomised to receive rasagiline 1 or 2 mg/day or placebo for a certain period, followed by rasagiline application in general. The relative weak degree of motor improvement was comparable to that seen for selegiline in the DATATOP study [3]. Patients who received 1 mg rasagiline during the whole study interval had less functional decline, as assessed by the Unified Parkinson’s Disease Rating Scale (UPDRS), than patients who received placebo initially for a certain period [4,5]. All these results support a certain disease modifying or progression slowing effect of MAO-B-I, but curative therapeutic approaches for PD are still elusive.

### Dopamine agonists (DA)

Ergoline and non ergoline DA act directly on postsynaptic dopamine receptors without the need for metabolic conversion to dopamine, storage and release in degenerating nigrostriatal nerve terminals. In addition, DA decrease endogenous dopamine turnover. All DA show a limited tolerability due to predominant nausea and dizziness in the initiation period. Therefore DA titration is performed in a slow and cautious manner. Additional temporary intake of the world wide only partially available dopamine antagonist domperidone against nausea and midodrine due to the onset of an orthostatic syndrome limit these side effects. Loss of appetite, sleepiness and/or oedema may also occur and reduce compliance of DA intake. The availability of various DA enables the switch from one DA to another to test the individual optimum tolerability and response (Table 2). Transdermal DA delivery with the rotigotine patch is also efficacious. Local allergic skin reactions appear immediately or after several months. This suggests a delayed allergic immune reaction triggered with a still unknown long lasting immune reaction cascade [6].

**Table 2:** Current available, approved dopamine agonists

compound	dose range	administration	half life
bromocriptine	10 - 50	oral, t.i.d.	3 – 6
lisurid	0.2 - 3	oral, 12 i.d.	2
pergolid	0.5 - 6	oral, t.i.d.	6-8
dihydro-α-ergocryptine	20 - 120	oral, t.i.d.	16
cabergoline	0.5 - 4	oral, o.i.d.	63
rotigotine	2 - 16	patch, t.i.d.	24
Pramipexol [retarded release]	0.25 - 4.5	oral, t.i.d.[o.i.d. – b.i.d.]	8 [24]
Ropinirol [retarded release]	4 - 24	oral, o.i.d. [o.i.d. – b.i.d.]	6 [24]
Piribedil retarded release	50 - 250	oral, t.i.d.	21

Dose range is given in mg, only approved dopamine agonist with oral or transdermal administration are listed, plasma half life is given in hours, 10 mg bromocriptine correspond to approximately 400 mg LD/DDI.

#### Why are non ergoline DA now preferred?

Ergoline DA induced fibrosis is the most serious and sometimes life threatening condition with delayed appearance and diagnosis due to insidious onset and onset of symptoms after several years of well tolerated DA treatment. Possible mechanisms are an idiosyncratic immune response with the drug acting as a hapten or an altered function via long term 5-HT stimulation with a consecutive induction of the key mediator of fibrosis, the transforming growth factor-β 1 [7]. However, these rarely occurring phenomena induced serious warnings on long term ergoline DA intake i.e. pergolide, cabergoline [6].

#### The advantage of slow release DA tablets

Slow release non ergoline DA are available now. These formulations showed a better tolerability and efficacy on additional non motor symptoms, i.e. sleep, in clinical trials. In daily practice, their handling is also better, since patients are mostly asked to take the drug one time daily only, which improves compliance (Table 2). Mixed results exist in terms of efficacy of the slow release formulation in comparison with the conventional formulation. Ropinirole slow release showed superior effect to normal released ropinirole [8]. Pramipexole slow release only demonstrated non inferiority to normal release formulation [9].

### NMDA-Antagonists

NMDA – antagonists, i.e. amantadine, improve motor symptoms by an indirect dopamine stimulating effect, particular infusions of amantadine sulphate are efficacious [10]. Clinical trials also showed a certain beneficial effect on motor complications (MC), i.e. involuntary movements or dyskinesia [11]. However there is need for further confirmatory placebo controlled trials [12]. Amantadine may support onset of hallucinations, psychosis, insomnia and oedema [10,12].

### Anticholinergics

Anticholinergics are nowadays rarely used due to peripheral and central side effects, like mouth dryness, constipation, miction problems, tachyarrhythmia, delirium and dementia. Sometimes one may consider using anticholinergic compounds for treatment of severe tremor in younger PD patients without any cognitive disturbances [13].

### Start of LD is a milestone

All the above mentioned compounds for dopamine modulation or - substitution improve motor behaviour in PD patients only to a certain extent. Therefore the introduction of LD is a necessity at a certain moment, which is looked upon as an essential milestone in each life of a PD patient. LD is the most efficacious and best tolerated compound for the control of motor symptoms in PD patients.

#### Basic principles of LD administration

**Figure 1:**
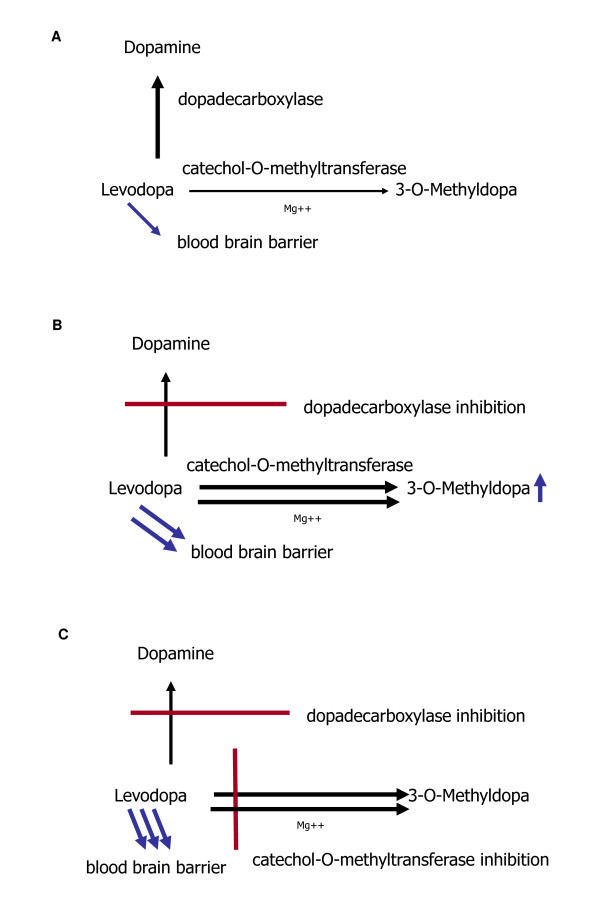
Types of LD degradation without (1 A) and with (1 B, 1 C) inhibition of LD degrading enzymes.

The introduction of LD was a pharmacological breakthrough in the treatment of PD. Initially LD was given as an infusion, then in oral form without inhibition of LD degrading enzymes (Figure 1 A). Oral LD application was later combined with DDI [14,15]. This pharmacological principle of enzymatic inhibition of LD metabolism reduces the peripheral degradation of LD to dopamine. Therefore, plasma half life of LD increases, which results in a better efficacy of the compound (Figure 1B). DDI such as Benserazide (BE) and Carbidopa (CD) do not cross the blood–brain barrier. Addition of DDI to LD allows for a four- to fivefold oral LD dose reduction [16]. As a result, the frequency of LD-related peripheral side effects, i.e. nausea and vomiting, lowers. LD absorption and efficacy can be delayed or diminished by amino acids in protein meals. Proteins may interact with the active large amino acid transporter of the gastrointestinal tract and of the blood brain barrier [17,18].

#### Supplementation of LD/DDI application with inhibitors of catechol-O-methyltransferase (COMT)

Inhibition of LD degradation by combination with a DDI supports LD metabolism via the enzyme COMT (Figure 1 B). As a result, an increased synthesis of the LD metabolite 3-O-methyldopa (3-OMD) occurs. Blocking of COMT activity further reduces peripheral LD degradation, as it prolongs plasma half life of LD and elevates delivery of LD to the brain. Moreover the peripheral LD degradation to 3-OMD is reduced. Experimental and clinical outcomes confirmed the efficacy of this therapeutic principle with peripheral dual inhibition of both main LD metabolizing enzymes (Figure 1 C) [16].

#### Oral LD/DDI long term application and progression of PD

Following the introduction of oral LD/DDI treatment, a debate was soon started on the saga of LD neurotoxicity. This discussion based on the clinical observation of fluctuations of movement as long term consequence of LD/DDI treatment and on outcomes of experimental animal – and cell culture studies [19]. As consequence, the Earlier vs Later L-DOPA (ELLDOPA) trial was designed to answer whether LD is harmful or not [20]. The ELLDOPA trial was the first double blind trial, which compared the therapeutic efficacy of LD/CD in three different daily dosages of 150 mg, 300 mg or 600 mg with placebo treatment according to the guidelines of good clinical practice in PD patients. Additionally functional brain imaging with the ^123^ J]-β-CIT-SPECT was employed as biomarker to evaluate the integrity of the nigrostriatal system during LD treatment and to demonstrate a PD accelerating effect of LD. The ELLDOPA trial produced conflicting results. Treatment with LD/DDI was associated with a significant increase in declining rates of ^123^J]-β-CIT imaging marker uptake over nine months compared with placebo, a finding consistent with a toxic effect of higher LD dosages. Clinical evaluation, however, showed that patients on LD/DDI treatment had better UPDRS scores compared with placebo administration after two weeks of washout. This would, in contrast, be indicative of a protective, benign PD modifying effect of LD. The simplest explanation for this effect is that the washout period was too brief to eliminate the symptomatic benefits of LD despite the short plasma half life of the drug. An alternative perhaps clinical more likely hypothesis may be that LD maintained body function and prevented onset of secondary long term changes and adaptation occurring after the manifestation of PD. This consideration may allow the more general conclusion that the clinical results of the ELLDOPA trial also support the concept of an early as possible diagnosis of PD and a subsequent immediate initiation of optimum treatment, as shown in the TEMPO- and the ADAGIO-study [5,21].

#### Clinical efficacy of COMT-inhibition in combination with LD/DDI

Numerous phase II, III and IV trials demonstrated that COMT-inhibition with entacapone (EN) or tolcapone given as extra tablet improves efficacy of LD/DDI [22]. A further important trial on the efficacy of COMT inhibition with EN was the FIRST-STEP (Favorability of Immediate-Release Levodopa/Carbidopa vs STalevo Short-Term comparison in Early Parkinson’s disease) study. It aimed to compare the efficacy of these two different modes of LD application, the conventional LD/CD administration versus LD/CD with the COMT-inhibitor EN in one tablet (LD/CD/EN) in early PD patients with a need for LD therapy. This multicenter, double-blind, randomized, parallel-group study administered a fixed oral LD dose of 300 mg/day, distributed as 100 mg LD doses three-times daily at 5-hour intervals to 424 PD patients. In this 39 weeks lasting study, the PD patients in the LD/CD/EN arm performed significant better than the ones in the LD/CD treated cohort after week four throughout the remaining course of the study. This was found when the sum scores of the UPDRS part II (activities of daily living) and UPDRS part III (motor examination) were compared as main primary outcome at the remaining study visits. The FIRST-STEP trial demonstrated that LD/CD was inferior to LD/CD/EN treatment [23]. Thus it confirmed the known additional LD/DDI efficacy enhancing effects of EN, when given as extra tablet, to an existing LD/CD regimen in treated PD patients, which was proven by pharmacokinetic investigations before [24].

#### Safety and tolerability of COMT-inhibitors

Phase III studies and post marketing surveillance showed the safety, tolerability and efficacy of LD/DDI combination with EN even with co-administration of selegiline, dopamine agonists and antidepressants. The most common observed side effect was harmless discoloration of the urine. A further non dopaminergic adverse event of COMT inhibition is diarrhoea sometimes occurring even up to two to four months following treatment initiation. This may be due to the inhibition of 5-HT metabolism in the gastrointestinal tract, which causes an increase of gastrointestinal motility in some PD patients [25]. Centrally acting, stronger direct EN competitor tolcapone was temporarily withdrawn due to reports on serious hepatic reactions with development of severe, sometimes even fatal, hepatic disease as well as possible occurrence of rhabdomyolysis and neuroleptic malignant-like syndrome. Nowadays one assumes that patients with mutations in the UDP-glucuronosyltransferase 1A9 gene, which leads to defective glucuronidation activity, may predispose for tolcapone induced hepatotoxicity.

#### Regulatory affairs

The discussion on the liver toxicity of tolcapone with a demand for liver function tests on regular basis still bias the preference for EN intake. An additional negative criterion for tolcapone use is the need for a previous failed response or intolerance of EN intake.

#### Oral intake of available COMT-Inhibitors

Tolcapone therapy asks for the additional intake of three tablets to a consisting LD/DDI regime only. EN, given as an extra tablet, should be combined with every LD/DDI intake with an upper limit of a daily dose of 1600 mg EN. This may increase the number of tablets and therefore may reduce the compliance. This disadvantage of EN therapy was improved with the introduction of the triple combination LD/CD/EN, which allowed to reduce the number of tablet intake and provided a smaller pill size. This further eased swallowing and favoured patients’ acceptance [25].

#### Reasons for delayed LD treatment initiation in PD patients

Current guidelines suggest a delay of LD use in PD patients as long as possible. Particularly, if one may assume a further long lasting necessary treatment of PD due to a relative young biological age. Avoidance of LD implementation is known to postpone onset of MC. MC limit quality of life and cause caregiver burden considerably [26]. There is study based evidence that in the long term outcome quality of life does not differ much depending of which drug, levodopa or pramipexole, has been used for treatment initiation. On the one hand LD is associated with more MC. On the other hand pramipexole causes more acute side effects, i.e. nausea, and less motor control [27].

#### Causes for the onset of MC

MC are fluctuations of movement behaviour dependent, which is called predictable, respectively independent, which is classified as unpredictable, on previous dopaminergic drug intake. MC are looked upon as a clinical milestone, which indicates an advanced stage of PD. MC are predominantly associated with oral LD/DDI treatment, as LD has a short plasma half life. Accordingly peaks and troughs of LD plasma levels appear. Following the LD transport into the brain and the conversion to dopamine in presynaptic neurons, fluctuations of dopamine brain concentrations generate MC by irregular, not continuous stimulation of nigrostriatal postsynaptic dopamine receptors. Loss of presynaptic dopaminergic autoreceptor function and other compensating abilities to avoid not physiologic high dopamine concentrations in the synaptic cleft are currently looked upon as the main central cause for MC appearance. Onset of MC is predominantly looked upon as the clinical consequence of the induction of frequent alternating postsynaptic dopamine receptor stimulation with further downstream intracellular changes in neuronal nigrostriatal cells, which regulate motor behaviour [26]. Continuous stimulation of these postsynaptic dopamine uptake sites may delay onset of MC, which often appear in combination with a wide array of non motor symptoms [28]. However peripheral components of drug intake, like compliance, which is a problem in many PD patients, or absorption of the applied compound often in combination with other drugs may also influence the occurrence of MC.

#### Consequences of MC in clinical practice

Generally, intervals and intensity of MC may differ from day to day. They may be brief or long, lasting between several minutes or hours. These fluctuations of movement behavior cause patient disability, embarrassment, frustration [28]. The ELLDOPA trial confirmed the general view, that the higher the daily administered LD dose was, the more frequent MC were observed (Table 3). Their occurrence rate was equal to placebo in the 150 mg daily LD dose treated cohort, but rose up to 3-fold in the 600 mg daily dose treated group [21]. Therefore generally, fear of MC limits the long term value and the patients’ acceptance of LD/DDI intake.

**Table 3:** Frequency of MC in the ELL-DOPA trial (%)

	LD/CD	LD/CD	LD/CD	
	150 mg	300 mg	600 mg	Placebo
dyskinesia	3.3	2.3	16.5	3.3
wearing off	16.3	18.2	29.7	13.3

#### MC: Wearing off

PD patients tend to experience fluctuations of movement with progression of the disease. They switch from ON to OFF and vice versa. The ON state is characterised by good movement behaviour. OFF is associated with temporary onset of the cardinal motor symptoms. When this reappearance of motor symptoms indicates the decreasing efficacy of the last dopaminergic drug intake before the next one, they are described as wearing-off.

#### EN and the treatment of wearing off

Both studies, FIRST-STEP and ELLDOPA, provided also some interesting findings regarding the onset and frequency of wearing off in PD patients (Tables 3 &4). In the FIRST-STEP trial, the number of monitored wearing off was higher in the LD/CD - compared with the LD/CD/EN treated PD patients (Table 3). The frequency of noted wearing off phenomena was rather low in relation to the size of the study population and the short observation interval. Therefore this did not turn out as statistical relevant in the investigated early PD patients, who were probably in the honeymoon period of LD application with good tolerability and efficacy. This may indicate that EN prevents onset of wearing off [23]. In the ELLDOPA trial, the number of observed wearing off increased with higher LD/CD dosing (Table 3). Therefore one may assume that EN in combination with LD/CD not only improves but also may prevent onset of wearing off phenomena. Accordingly, pharmacokinetic studies showed that EN supplementation to LD/CD avoids troughs, elevates half life of LD and thus contributes to more stable plasma levels after twice administration every three hours. The prolongation of plasmatic LD half life was even shown after one time application. All these outcomes contribute to prevent and to improve wearing off phenomena. The latter feature was the reason for approval of EN, but the value of EN supplementation to LD/CD in the treatment or prevention of other kinds of MC such as dyskinesia still remained unsolved.

**Table 4:** Rate of MC in the FIRST-STEP trial (%)

	LD/CD/EN	LD/CD	Total	
dyskinesia	2.7	4.2	3.5	week 39
wearing off	8.8	12.0	10.4	week 39
dyskinesia	5.3	7.4	6.4	at any study visit
wearing off	13.9	20	17	at any study visit

#### MC: Dyskinesia

Unwanted, abnormal involuntary movements are termed as dyskinesia. They develop as a complication of dopaminergic stimulation. Dyskinesia can occur during both ON and OFF intervals. Classification of dyskinesia is generally performed in relation to the timing of LD dosing. ON dyskinesia appears either during the period when patients are obtaining maximal relief from their motor symptoms. Then they are looked as peak-dose dyskinesia. The maximum plasma LD level following intake may cause peak-dose dyskinesia as the most common form of these kind of involuntary movement behaviour. They may be also biphasic, occurring soon after LD is taken and as the patient is beginning to turn ON and again when the LD effect is wearing off and the patient is beginning to turn OFF. They are mostly absent when the LD dose is having its maximal effect. As the disease progresses, patients may develop dyskinesia throughout the whole ON time. The risk of developing dyskinesia has been associated with a number of clinical factors. The severity of PD, the dosage and duration of LD therapy, low body weight and a younger age of the PD patient are currently believed to be among the variables that best predict the development of dyskinesia [26].

#### Continuous introduodenal LD/DDI treatment

Initially LD is well tolerated and provides no MC. This is called the honeymoon period of LD therapy, followed by the insidious onset of MC and associated non motor features, when the drug efficacy vanes. Therefore pharmacologic strategies were necessary to prolong the half life of LD, as a more continuous LD delivery to the brain helps to prevent and improve predominantly LD/DDI associated MC [29]. The efficacy of this concept and the importance of not fluctuating LD brain delivery were convincingly shown by duodenal LD infusion systems, as PD patients suffering from severe MC experienced an enormous deterioration of the intensity and frequency of MC due to the stable LD plasma concentrations. But performance of duodenal LD infusions is a rather complex and an expensive technique with a considerable demand for caregiver burden [30].

#### Preventive treatment concepts for dyskinesia

Studies in drug-naive animal models of PD have shown that continuous dopaminergic stimulation is associated with reduced incidence and severity of dyskinesia compared with pulsatile administration. Continuous dopaminergic stimulation due to a more continuous delivery of dopaminergic drugs to the brain may be achieved through the administration of transdermal dopamine agonist administration, retarded release dopamine agonists or intra-duodenal LD application or to a lesser extent by administering frequent doses of LD/DDI, so called LD fractionation, with or without a COMT inhibitor.

#### COMT inhibition and dyskinesia onset

Addition of COMT-inhibitors to a LD/DDI regimen without concomitant individual adaption of LD/DDI intake by extension of dosing intervals or reduction of LD/DDI dosage may induce dyskinesia. Pharmacokinetic and clinical trials on the effects of repeat COMT intake showed, that the addition of COMT-inhibitors increases peak levels and thus the amount of plasma LD, which is delivered to the brain. Both may support onset of dyskinesia. Therefore it is often necessary in clinical practice, to modify oral LD/DDI intake, when a COMT inhibitor is additionally introduced even in PD patients with wearing off. More generally these pharmacokinetic findings in PD patients and healthy volunteers also suggest, that LD/CD/EN as first line LD formulation may be more appropriate, as LD/CD/EN provides less fluctuating LD plasma levels, as shown by pharmacokinetic trials. Moreover from the clinical point of view, LD therapy is less complex, when LD/CD/EN is introduced as first line LD formulation, if tolerated, in PD patients. This approach circumvents the above mentioned COMT inhibitor related complex adaption of LD intake following the onset of wearing-off phenomena. The ELLDOPA study confirmed that LD dose is a factor in causing dyskinesia and that these can even develop as early as 5–6 months after treatment initiation. Patients receiving 600 mg/day experienced significantly more dyskinesia than patients receiving placebo, 150 or 300 mg/day (p < 0.001). The FIRST-STEP trial reported a not significant tendency towards a lower number of observed dyskinesia in the LD/CD/EN treated arm compared with the LD/CD treated PD patients (Table 4) [23]. The study was not powered to demonstrate this effect. An experimental animal trial showed lower frequency and less intensity of dyskinesia, when a treatment with LD/DDI with the COMT inhibitor EN, given four times daily, was started right from the beginning [31]. A further confirmatory result should be provided by the outcomes of the STRIDE-PD study (STalevo Reduction In Dyskinesia Evaluation), however this trial failed [32]. Nowadays it is known that repeat EN dosing may increase of C_max_ values of LD in plasma, which in turn augments the risk for onset of dyskinesia. These pharmacokinetic characteristics of LD plasma behaviour in the context with EN supplementation to a LD/CD regimen was underestimated respectively not known, when STRIDE-PD was designed. However the design of STRIDE-PD did not allow prolonging of dosing intervals or dose reduction after initial EN supplementation to an existing LD/CD regimen [22,33].

#### Failed earlier preventive strategies for MC onset by oral LD application

Earlier, LD/DDI application with oral slow release formulations were developed and tested as an alternative to provide more constant LD plasma behaviour. Clinical studies showed a reduced efficacy of these retarded release LD/CD formulations in comparison with conventional LD/CD tablets, when the same oral LD dosage was administered. Moreover they did not delay onset of MC. However one must consider that these trials did not evaluate MC with too much detail [34,35].

### Current effective therapy regimen for alleviation of MC: Deep brain stimulation and infusion techniques

#### Deep brain stimulation

Both sided deep brain stimulation (DBS) of the subthalamic nuclei reduces the dosages of dopaminergic drugs, improves motor symptoms and motor complications, but not speech, gait or postural dysfunction. This method may cause social adjustment problems, depression and cognitive disturbances. Depression might occur in the postoperative phase but there is no clear evidence for DBS induced depression in the long term. When respecting the inclusion criteria for DBS only distinct cognitive functions like decline in word fluency can be related to the DBS itself whereas general cognitive deterioration may rather be related to disease progression itself [36-40]. Nevertheless careful selection of PD patients without psychiatric and cognitive symptoms is essential.

### Infusion techniques

The present infusion systems administer dopaminergic drugs continuously. They provide benefit on MC. Their complex application and titration modes need to be simplified. Local inflammatory subcutaneous noodles at the subcutaneous DA administration site may appear. Apomorphine is mostly administered in combination with other oral antiparkinsonian drugs. The duodenal LD/CD pump system still suffers from hardware problems, i.e. at the duodenal administration site. LD/CD is given in high dosages, mostly in monotherapy. Rarely, axonal polyneuropathy occurs, which is often associated with vitamine B deficiency [41,42]. Both systems are expensive [30,43].

## Dopamine substitution and non motor symptoms

Dopaminergic drugs improve the mainly dopamine related motor symptoms in PD. They only partially influence non motor symptoms [1,44]. The drug therapy of non motor features is complex due to possible occurrence of drug interactions, interference with side effects and induction of altered pharmacokinetic behaviour of dopamine substituting compounds. But drug treatment of non motor symptoms in PD gains more and more interest. Thus trials showed a moderate positive effect of the dopamine agonist pramipexole on depression in PD [45]. These investigations reflect a changing attitude from the focus on the effects of centrally acting compounds on the dopaminergic neurotransmission towards a more general view of PD with consideration of neuropsychiatric features under long-term aspects. However, PD drugs themselves may also contribute to onset of non motor symptoms, such as visual hallucinations. This complicates the differentiation from the disease process itself.

## Therapy of non motor features

Treatment of non motor symptoms in PD is important. Course of PD, expression of motor - and non motor symptoms, efficacy and tolerability of therapeutic interventions vary from one patient to another in clinical practice. This asks for an individualized drug regime with repeated control and titration by the treating physician in close cooperation with the patient and his caregiver [44].

### Sleep disturbances

Up to 75% of PD patients complain of insomnia particularly during long-lasting sleep at night. Reduced mobility related to akinesia may cause lowered turning movements during bedtime. Onset of dystonic cramps or pain due to increased stiffness may disturb sleep. Bladder dysfunction in combination with polyuria may cause further sleep complications [44].

#### Therapy

Sleep-onset- as well as sleep maintenance insomnia is often induced by dopamine deficiency during night time. As consequence symptoms like nocturnal akinesia, nocturia and even periodic limb movements may occur. Relief may be provided by intake of slow release LD/CD (BE) preparations, their equivalent LD/CD/EN formulations late in the evening and intake of slow release dopamine agonists, respectively the rotigotine patch [46]. A further approach for more advanced PD patients may be to take fast release or conventional LD/DDI formulations during waking periods during the night. Since a further frequent psychopathological feature of PD, depression, may cause sleep disturbances, intake of sedative antidepressant compounds is often recommended. Retarded Melatonin and gabaergic drugs are also useful. Zolpidem with its short half life or Zopiclon with a longer half life are efficient drugs dependent on the kind of sleep disturbance. Benzodiazepines especially in the elderly may sometimes cause paradoxical reactions, such as agitation. Moreover they may induce dependence. Therefore they should be used cautiously. Sedative atypical antipsychotic agents, like clozapine or quetiapine, should be given in the case of vivid dreams, in pre-psychotic states and psychosis [44,47]. Sleep fragmentation, avoiding of alcohol, caffeine, nicotine etc. are essential not for additional drug intake demanding, initial essential treatment options.

### Sleepiness

Dopamine substitution itself may cause sleepiness and fatigue sometimes combined with sudden so-called “sleep attacks”during the day. PD patients complain of these sedative adverse events following the direct administration of dopamine substituting drugs [44].

#### Therapy of daytime sleepiness

Methylphenydate, dextroamphetamine, pemoline, modafinil and amantadine are known to improve wakefulness and vigilance. Therefore these compounds may provide also a certain benefit on daytime sleepiness and sleep attacks [48].

## Depression

Apathy, anxiety, panic attacks as features of depression appear not only in early stages but also in the further course of PD frequently. A bad motor situation or motor complications induce physical impairment and therefore contribute to onset of depressive mood, as quality of life is lowered. These reactive components in turn facilitate onset of endogenous components of mood disturbances, which are associated with the progressing chronic neurodegeneration in non dopaminergic neurotransmitter systems [44].

### Therapy of depression

Concomitant psychotherapeutic and behavioural interventions are essential. Choice of the antidepressant drug should consider its efficacy on the depression itself, its long-term effect on the motor system, its potential for interactions with the concomitant PD drugs, its specific and non-specific drug side effects with regard to other non motor symptoms, i.e. cognitive function, micturition, salivation, orthostasis.

Motor complications may also trigger anxiety and panic attacks in predisposed PD personalities. Up to 64% of PD patients additionally suffer from mood disorders, which also impact their spouses with up to 34%. Effective antidepressant compounds are sedative (i.e. mirtazapin) or more activating (i.e. citalopram) antidepressants or compounds like bupropion, which influence noradrenergic or dopaminergic pathways, or nefazodone, trazodone and venlafaxine, which impact neurotransmission of serotonin and norepinephrine. This impact on norepinephrine turnover is under suspicion to improve orthostatic syndrome, however convincing trials are not available. Tricyclics should be avoided in particular due to their anticholinergic properties, as they may facilitate onset of cognitive disturbances in the long term in the elderly PD patients [44]. One small study even reports more responders, when tricyclics are employed for therapy of depression in PD [49]. Another trial however emphasizes the lower tolerability of desimipramine in comparison with citalopram and placebo [50].

## Cognition in PD patients

Cognitive slowing and apathy may respond to optimum dopamine substitution, as they are triggered by parts of the dopamine innervated mesolimbic system. The borders towards a disturbed memory function are fluent. Clinical initial symptoms of dementia are deficits of attention, cognitive slowing, executive, visual spatial and memory dysfunction, an increased vulnerability for onset of illusions or optic hallucinations by centrally acting drugs. However a clear distinction between apathy, depression and dementia based on clinical observation only is difficult. A distinct cholinergic deficit in PD patients is looked upon as the main cause from the neurochemical point of view [51].

### Drug therapy of cognitive problems

Inhibitors of acetylcholinesterase improve symptoms of dementia in PD patients and allied conditions. Predominant open label smaller trials with donepezil and rivastigmine demonstrated a better cognitive function in various kinds of patients with impairment of motor and cognitive function, whereas memantine only showed a limited effect. Rivastigmine, an inhibitor of both acetylcholinesterase and butyrylcholinesterase, produced a moderate but significant improvement in global ratings of dementia, cognition with measurements of executive functions and attention, and neuropsychiatric behavioural symptoms among patients with dementia associated with PD [52]. This effect is in particular pronounced in PD patients with a homocysteine elevation [53]. This effect of elevated homocysteine level associated therapy response was also seen with memantine [54].

## Psychosis

The chronic neurodegenerative process and the concomitant chronic dopaminergic stimulation predispose for onset of psychosis probably triggered by an imbalance between the dopaminergic and glutamatergic system. Vivid dreams, fear, predominant optic illusions, anxiety, paranoia, hallucinations and loss of sleep are initial clinical signs similar to delirium [44]. Occurrence of symptoms may be predisposed by the premorbid personality structure.

### Symptomatic causes of psychosis

Generally, nearly each PD drug may cause psychosis in particular in combination with dehydration. First hydration and then careful reduction of dopaminergic drugs with subsequent reduced motor function are the most useful clinical means to treat and avoid exacerbation of psychosis. Treatment of additional concomitant infections or other general diseases, which may induce psychosis and/or delirium, is often necessary. Reduction of medical, social or other kinds of stress, i.e. surgery, change of environment, are further useful means.

### Drug treatment of psychosis

Since classical neuroleptics, like butyrophenones or phenothiazines, increase extrapyramidal symptoms, preponderantly atypical neuroleptics are used for prevention and treatment of psychotic symptoms in PD. The atypical antipsychotic agent clozapine is well tested in clinical trials, shows additional sedative and tremorlytic components and prevents recurrence of psychosis. However rare induction of leucopenia with resulting necessary blood counts on a regular basis and sometimes initially occurring transient subfebrile temperatures limits its use. Since clozapine is metabolized via CYP1A2, CYP3A4, CYP2C19 and CYP2D6, increase of drug levels and/or intoxications may appear in combination with drugs, which have these metabolic pathways, i.e. SSRIs, like paroxetine or fluvoxamine, phenothiazines and related compounds [44]. Quetiapine, which shares a certain structural similarity to clozapine, has the same antipsychotic efficacy. But the sedative components are not so pronounced. This is a drawback in the case of acute psychosis treatment. However this drug has a distinct lower muscarinergic potency in comparison with clozapine [55]. Quetiapine is also known, to enhance dopamine enrichment in the prefrontal cortex, therefore it may reduce cognitive slowing [56]. Accordingly, several open trials even reported improvement of cognitive function even in PD patients, who did not respond to clozapine. This is an effect that may essentially reduce caregiver burden. Therefore quetiapine appears to be more suitable for the long-term use of antipsychotics for treatment or even prevention of onset of psychosis, which is often performed in clinical practice in order to achieve a better motor condition in advanced PD patients. However quetiapine failed in clinical trials in these indications in PD patients. Therefore it is not regarded as useful as clozapine by evidence based medicine recommendations, whereas clinicians widely use this compound sometimes even off label for the treatment of their PD patients in some countries [57]. Further treatment options are sedative and less cheaper antipsychotic drugs with a low dopamine receptor blocking potential, like the buthyrophenones melperone or pipamperone. However these compounds also worsen the motor situation in the long run similar to other so-called atypical high potential antipsychotic compounds, like olanzapine and risperidone. Other antipsychotic typical drugs, like the butyrophenone haloperidol or the thioxanthene flupentixol, should be avoided due to their high affinity to postsynaptic dopamine receptors.

## Cyclic mood disorder with hypomania or manic psychosis

Manic episodes or psychosis are often associated with dopamine dysregulation syndromes, more rarely they appear following deep brain stimulation. Treatment options are atypical neuroleptics, i.e. clozapine and/or quetiapine. An alternative may be reduction of dopamine replacement therapy however this will worsen the motor situation [58].

## Dopamine related impulsive-compulsive disorders

Noticeable problems of the impulsive-compulsive spectrum do not occur so frequently, but their onset may be related to PD itself, to the pharmacological management of PD or to both. These diseases comprise dopamine deficiency syndrome with immediate reward seeking behaviour, dopamine dependency syndrome with addiction and dopamine dysregulation syndrome with both addiction and stereotyped behaviour. Additionally impulse control disorders, like pathological gambling, compulsive shopping, binge eating, punding and hypersexuality, are observed. These changes are especially seen in PD patients with young age of onset, higher dosing of dopamine substituting compounds, depression, recreational drug or alcohol abuse, and high novelty seeking personality traits. The role of dopamine in the mesolimbic brain structures points out, that this amine is not only involved in voluntary movement control. Thus dopamine also plays an essential role in the reward system and the modulation of behaviour. Consequently most if not all drug-naive PD patients suffer from dysphoria, leading to mild immediate reward seeking behaviour. Some patients under dopamine substitution demand for the intake of increasing quantities of LD, above those required to adequately treat motor features of PD. Therefore they show all characteristics of a dopamine dependency. These patients may also develop plastic changes in the striatal matrix, which may support onset of dyskinesia, caused by extracellular fluctuations of striatal dopamine levels due to pulsatility of dopamine replacement. As soon as these changes are also seen in the striatal striosomes, in the framework of a dopamine dysregulation syndrome, stereotyped behaviours, like punding, may occur. Thus impulse control disorders may be regarded as adverse side-effects of stimulation with dopamine [59].

### Treatment concepts

Treatment of impulse control disorders is associated to the underlying pathophysiology. In the case of dopamine deficiency, dopamine replacement will help. Psychosocial strategies will help to improve the multifactorial dopamine dependency and dysregulation syndromes, respectively addictive behaviour. Stereotyped behaviour, like punding, may be covered by continuous dopaminergic receptor stimulation. In case of i.e. drug induced or extrinsic impulse control disorders, a therapy concept may be the reduction or the replacement of dopamine receptor agonists [59]. In case of pathological gambling, this may be associated with other abnormal actions such as pathological shopping, hoarding and hypersexuality. The incidence of these syndromes varies widely from study to study but may be up to up to 13.6% of users of dopaminergic agents [60]. Recognition of this problem has led drug regulatory agencies to add precautions concerning pathological gambling to official drug information for the entire class of PD drugs. The literature is not entirely consistent and opinions differ greatly, but the combined dopamine D2/D3 receptor agonists pramipexole and ropinirole may be especially likely to be associated with pathological gambling. However the precise nature of the relationship is unclear and may also be related to the widespread use of these two compounds. It must be emphasized, that clear treatment strategies are not available yet. However these syndromes gain more and more attention.

## Autonomic failures

Autonomic dysfunction may considerably reduce quality of life, in particular in advanced PD stages [44]. Treatment options of autonomic features seborrhea, hyperhidrosis, orthostatic syndrome, excessive salivation, bladder dysfunction, gastrointestinal disturbances are given in Table 5.

**Table 5:** Treatment options of autonomic failures in Parkinson’s disease

symptom	treatment options
salivation	belladonna compounds, anticholinergic drugs, glycopyrrolate, botulinum toxin (off label use), 1% atropine eye solution (off label use)
seborrhea	soaps, shampoos
hyperhidrosis	botulinum toxin
constipation	Various kinds of laxatives, sufficient hydration, fibers, prucaloprid (Reselor®), macrogel (Movicol®)
gastrointestinal motility	Domperidone, prucaloprid (Reselor®) (off label use !)
bladder dysfunction	genneral approach: reduced fluid intake at night is sometimes helpful. parasympatholytics, imipramine, Fesoterodinfumarat (Toviaz®) Darifenacin (Emselex ®), botulinum toxin (off label use !) (in case of imperative urgency due to overactive bladder syndrome [detrusor hyperreflexia or overactivity]) optimum dopaminergic drug titration (in case of frequent and/or involuntary urinary incontinence due to uninhibited contractions of the detrusor muscle) distigmine bromide, reduction of anticholinergic drugs, (in case of detrusor hyporeflexia [underactivity])
sexual dysfunction	sildenalfil, oral apomorphine, alprostadil, psychotherapy
orthostatic syndrome	patient education, non pharmacological interventions, midodrine, fludrocortisone, yohimbine, droxidopa

## Pain and sensory symptoms

Pain is often proportional to the degree of motor dysfunction and may take the form of muscle cramps, stiffness, dystonia, radiculopathy and arthralgias. Sensory complaints appear as numbness, burning or tingling and occur at any stage of the disease. Sometimes they are independent of medications and the degree of motor deficits, but they may also appear in relation to motor fluctuations. Treatment options are local botulinum toxin applications, subcutaneous apomorphine injections, fast absorbed, soluble LD preparations and symptomatic pain relief with antirheumatic compounds or gabapentin, pregabalin in the case of additional painful polyneuropathy [44].

## Osteopenia and sceletal deformaties

Deformaties of feet and hands are common in PD. Coincident with rigidity, changes occur in the curvature of the spine. Initially mild scoliosis may occur, concave contra lateral to the affected side. Later kyphosis becomes prominent and supports onset of postural instability. Osteopenia may contribute to those deformities to a large extent. High prevalence of hip and other fractures in PD is not only due to increased number of falls because of problems of gait and postural instability, but also because of reduced bone mineral density due to a deficiency of 1,25-dihydroxyvitamine D, 25-hydroxyvitamine or an age related reduction of the amount of 1 α-hydroxylase. Therapeutic options are supplementation with calcium plus 1α-hydroxyvitamine D3 [44].

## Cure: Still a dream

Neuroregenerative transplantation - and curative growth factor trial outcomes disappointed. Future stem cell therapy is far from clinical testing. The essential problem of all these more regenerative therapeutic approaches is their specific focus on the dopamine system without consideration of altered non dopaminergic neurotransmitter balance in PD and the missing control of dopamine release to the nigrostriatal system in a physiologic manner. Therefore the transplantation trials in idiopathic PD patients failed. Onset of dyskinesia and cognitive disturbances, which hypothetically result from dopamine overflow after a certain interval, were observed in the clinic [61]. A further approach is gene delivery of the trophic factor neurturin via an adeno-associated type-2 vector. Trials are ongoing [62].

## Conclusions

PD patients do not only suffer from motor symptoms, but also from non motor features. Thus treating PD patients asks for an individual and holistic approach. Therefore therapy recommendations for PD patients only based on the so-called evidence based medicine, which overemphasizes the value of clinical randomized placebo controlled studies according the guidelines of good clinical practice with its selected patient populations, are somewhat “foolish” and beyond reality in clinical practice [57]. Putative interactions between various applied drugs, recurrent appearance of non motor features and treatment of motor symptoms ask for complex therapeutic interventions and careful drug titration. Reduction of dopaminergic drugs and hydration may sometimes be more beneficial than addition of further compounds. This also avoids compliance problems and reduces individual differing tolerability of additional drugs for non motor symptoms. Long-term experience for the combined use of these supplementary drugs with dopaminergic agents is essential to achieve an improvement of quality of life and to prevent onset of drug side effects. Specialist knowledge of internal medicine, psychiatry and pharmacology is advantageous in order to avoid drug interactions with PD drugs and to guarantee adequate treatment. The choice of these supplementary agents must be considered very carefully and the start of additional application must be performed slow and cautious. Therapeutic options are often limited because of additional morbidity and concomitant drug therapy.

## Abbreviations

PD: Parkinson’s Disease; MAO: Monoaminooxidase; LD: Levodopa; DDI: Dopadecarboxylase inhibitor; TEMPO: a controlled trial of rasagiline in early Parkinson’s disease; ADAGIO: Attenuation of Disease Progression with Azilect Given Once-Daily; UPDRS: Unified Parkinson’s Disease Rating Scale; MAO-B-I: Monoaminooxidase B inhibitor; DA: dopamine agonist; MC: motor complications; BE: Benserazide; CD: Carbidopa; COMT: catechol-O-methyltransferase; 3-OMD: 3-O-methyldopa; ELL-DOPA Trial: Earlier vs Later L-DOPA trial; EN: Entacapone; FIRST STEP Study: Favorability of Immediate-Release Levodopa/Carbidopa vs STalevo Short-Term comparison in Early Parkinson’s disease) study; UPDRS part II: UPDRS part activities of daily living; UPDRS part III: UPDRS part motor examination; 5-HT: serotonine; STRIDE-PD study: STalevo Reduction In Dyskinesia Evaluation Study.

## Competing interests

The author received lectures fees, honoraria, grants etc. by GSK, TEVA, Lundbeck, Novartis, UCB, Archimedes in the past twelve months.
